# Serum vitamin D levels and risk of prevalent tuberculosis, incident tuberculosis and tuberculin skin test conversion among prisoners

**DOI:** 10.1038/s41598-018-19589-3

**Published:** 2018-01-17

**Authors:** Elisangela B. Maceda, Crhistinne C. M. Gonçalves, Jason R. Andrews, Albert I. Ko, Catherine W. Yeckel, Julio Croda

**Affiliations:** 1 Faculty of Health Sciences, Federal University of Grande Dourados, Dourados, Brazil; 2 School of Medicine, Federal University of Mato Grosso do Sul, Campo Grande, Brazil; 3 Division of Infectious Diseases and Geographic Medicine, Stanford University School of Medicine, Stanford, CA USA; 4 Department of Epidemiology of Microbial Disease, Yale School of Public Health, New Haven, CT USA; 5 Oswaldo Cruz Foundation, Salvador, Brazil; 6 Department of Environmental Health Sciences, Yale School of Public Health, New Haven, CT USA; 7 Oswaldo Cruz Foundation, Campo Grande, Brazil

## Abstract

Poor vitamin D status has been associated with tuberculosis (TB); whether poor status is cause or consequence of disease is uncertain. We conducted a case-control study and two nested case-control studies to determine whether vitamin D levels were associated with active TB, tuberculin skin test (TST) conversion, and risk of progression to the active TB in prisoners in Brazil. In multivariable conditional logistic regression, subnormal vitamin D levels (OR, 3.77; 95% CI, 1.04–13.64) were more likely in prisoners with active TB. In contrast, vitamin D was not found to be a risk factor for either TST conversion (OR, 2.49; 95% CI, 0.64–9.66) or progression to active disease (OR, 0.59; 95% CI, 0.13–2.62). Black race (OR, 11.52; 95% CI, 2.01–63.36), less than 4 years of schooling (OR, 2.70; 95% CI, 0.90–8.16), cigarette smoking (OR, 0.23; 95% CI, 0.06–0.79) were identified as risk factors for TST conversion. Risk of progression to active TB was found to be associated with cigarette smoking (OR, 7.42; 95% CI, 1.23–44.70). Our findings in the prison population show that poor vitamin D status is more common in individuals with active TB, but is not a risk factor for acquisition of latent TB or progression to active TB.

## Introduction

Tuberculosis (TB) is a major global public health problem, causing approximately 1.4 million deaths annually^1^. The World Health Organization estimates that one-third of the world’s population has latent TB^1^, of which approximately 10% will develop active disease^2^. Understanding which individuals will progress to TB and which will maintain lifelong immune control or achieve clearance, remains an important scientific question with substantial public health implications. This high-risk group also provides the opportunity to examine host factors that currently lack complete understanding in the disease pathway for active TB.

One of the prominent host factors that has been implicated in TB risk is vitamin D levels^3^. Nutritional deficiency, low exposure to sunlight due to spending only short periods of time outdoors, and seasonal changes resulting in reduced ultraviolet radiation type B (UVB) may be associated with subnormal vitamin D levels^4^. Vitamin D levels of <30 ng/ml, which are characterized clinically as either insufficient (29–20 ng/ml) or deficient (<20 ng/ml). Vitamin D is composed of a group of secosteroid molecules that are predominantly synthesized in the skin following exposure to solar radiation, resulting in vitamin D_3_ (or cholecalciferol), or obtained via dietary intake, resulting in vitamin D_2_ (or ergocalciferol) or D_3_^5^. Active vitamin D requires two hydroxylation reactions. The primary circulating form of vitamin D is 25(OH)D_3_ after vitamin D_3_ is hydroxylated in the liver. 25(OH)D_3_ is the precursor for the biologically active form of vitamin D, 1,25(OH)_2_D_3_, formed largely in the kidney. It is now known that immune cells (innate and adaptive), e.g., macrophages express enzymes to locally activate 25(OH)D_3_ to supra-physiological concentrations of 1,25(OH)_2_D_3_ for diverse functions. For example, it has been demonstrated that activated vitamin D is required for interferon-γ mediated antimicrobial activity of macrophages against *Mycobacterium tuberculosis*^6^.

Many studies have reported seasonality of TB disease incidence, leading to speculation that seasonal differences in vitamin D levels may play a role in TB infection or disease progression^7^. However, isolating the role of vitamin D from other seasonal confounders has been difficult. The majority of studies investigating the relationship between vitamin D levels and TB infection or disease risk have been cross-sectional in nature; as TB may cause micronutrient deficiencies, determining whether there is a causal relationship between vitamin D levels and TB risk remains challenging. One prospective study among household contacts in Spain found that vitamin D deficiency was associated with risk of TST conversion and incident disease among household contacts of TB cases^8^. Further evidence is needed to understand whether vitamin D levels are predictive of risk of incident TB infection and subsequent risk of disease.

Brazil is a medium TB-burden country, which has over 70,000 new cases TB diagnosed annually. Of the 526,000 new cases of TB reported between 2009 and 2014 in Brazil, 38,000 (7.3%) were diagnosed among prisoners^9^. In this population, the incidence of active TB is at least 30 times higher than that observed in the general population^10^. A number of structural and host determinants have been implicated in the high rates of TB infection and disease observed in prisons^11^. These include factors associated with increased transmissibility of the disease, such as overcrowding and ventilation^11^, as well host factors associated with both TB risk and incarceration, such as nutritional deficiency^12^, HIV infection, smoking^13,14^ and drug use^15,16^. Previous studies have identified low vitamin D levels among prisoners in correctional facilities, likely due to prolonged indoor incarceration with insufficient sunlight exposure or dietary sources of vitamin D^7^. Whether differences in vitamin D levels among prisoners are an important determinant of elevated TB risk is not understood.

The purpose of this study was to investigate the role of subnormal vitamin D in incident TB infection and disease risk among incarcerated individuals. We performed one case-control study and two, nested case-control studies among an observational cohort of Brazilian prisoners. We aimed to determine whether vitamin D levels were associated with three distinct features of TB disease: active TB, *M*. *tuberculosis* infection (tuberculin skin test (TST) conversion), and progression to active disease in this high-risk population.

## Results

### Risk factors associated with active TB

The frequency distribution of the variables and risk factors are shown in Table 1. We compared 24 TB cases and 48 matched controls without TB. The mean age of prisoners for the case-control study was 32 years (±7 years) among cases and 33 years (±8 years) among the controls (Table 2). Mean serum 25(OH)D_3_ level was significantly lower among the cases (27.7 ± 7.85 ng/mL) than controls (37.1 ± 8.94 ng/mL, p < 0.01; Table 2). Prisoners with active TB were more likely to have subnormal vitamin D levels (<30 ng/mL) when compared to healthy controls (18/24 [75%] vs 16/48 [33%], p = 0.004; Table 1). As shown in Table 1, there is a clear difference between cases and controls with active TB (p < 0.01). A greater difference existed among TB patients with vitamin D deficiency (3.5×), than those with vitamin D insufficiency (2.0×) when compared with controls. All cases had negative HIV serology and only two controls had positive HIV serology (Table 1).

**Table 1:** Frequency distribution of variables and risk factors. N, number; TB, tuberculosis; MS, Mato Grosso do Sul; TST, tuberculin skin test.

Variables	Case-control study			Nested case-control study			Nested case-control study		
	*Active TBN (%)*			*TST conversionN (%)*			*Progression to active TB N (%)*		
	Cases n = 24	Controls n = 48	p-value	Cases n = 30	Controls n = 60	p-value	Cases n = 24	Controls n = 48	p-value
Marital status (single)	12 (50)	19 (39)	0.40	17 (56)	29 (48)	0.43	11 (46)	20 (41)	0.70
Black race	17 (70)	29 (60)	0.10	22 (73)	34 (56)	0.05	19 (79)	27 (56)	0.04
Resided in MS	14 (58)	29 (60)	0.85	22 (73)	42 (70)	0.74	15 (62)	30 (62)	1.00
Less than 4 years of schooling	3 (12)	12 (25)	0.29	9 (30)	32 (53)	0.04	7 (29)	15 (31)	0.84
Cigarette smoking	15 (62)	23 (47)	0.19	10 (33)	31 (51)	0.09	20 (83)	24 (50)	0.008
Drug use during the last year	21 (87)	31 (64)	0.04	14 (46)	29 (48)	0.86	20 (83)	30 (62)	0.06
Alcohol use	17 (70)	27 (56)	0.35	11 (36)	30 (50)	0.17	16 (67)	31 (64)	1.00
Previous TB	3 (12)	3 (6)	0.35	1 (3)	2 (3)	1.00	3 (13)	3 (6)	0.39
HIV positive	0 (0)	2 (4)	—	0 (0)	0 (0)	—	0 (0)	1 (2)	—
BCG scar	20 (83)	44 (94)	0.17	22 (73)	48 (81)	0.38	20 (83)	43 (90)	0.45
Previously incarcerated	23 (95)	37 (77)	0.07	16 (53)	37 (61)	0.53	15 (63)	33 (68)	0.59
TST positive	14 (58)	23 (47)	0.46	0 (0)	0 (0)	—	8 (33)	24 (50)	0.36
Serum 25(OH)D level			<0.01			0.20			0.96
≥30 ng/mL	6 (25)	32 (67)		22 (73)	41 (68)		18 (75)	37 (77)	
20 to 29 ng/mL	13 (54)	13 (27)		7 (23)	6 (10)		3 (12)	6 (12)	
<20 ng/mL	5 (21)	3 (6)		1 (3)	13 (22)		3 (12)	5 (10)

**Table 2:** Mean and standard deviation of the continuous variables included. ^a^Significantly different from controls (t-test, p ≤ 0.05). SD, standard deviation; NA, not applied.

Continuous variables	Case-control study			Nested case-control study			Nested case-control study		
	*Active TB* (Mean ± SD)			*TST conversion* (Mean ± SD)			*Progression to active TB* (Mean ± SD)		
	Cases n = 24	Controls n = 48	*p*-*value*	Cases n = 30	Controls n = 60	*p*-*value*	Cases n = 24	Controls n = 48	*p*-value
Serum 25(OH)D level (ng/mL)	27.7 ± 7.85	37.1 ± 8.94	<0.01^a^	37.7 ± 11.93	34.5 ± 14.89	0.19	37.0 ± 14.83	37.5 ± 11.04	0.86
Age (years)	32.7 ± 7.95	33.0 ± 8.08	0.90	32.1 ± 6.87	31.1 ± 7.71	0.50	30.8 ± 8.90	31.9 ± 9.02	0.63
Serum albumin level (g/dL)	NA	NA	—	4.63 ± 0.43	4.28 ± 1.00	<0.01^a^	4.25 ± 0.83	4.25 ± 0.79	0.99
Body Mass Index (Kg/m^2^)	22.6 ± 26.0	26.0 ± 3.	<0.01^a^	24.9 ± 3.52	26.02 ± 4.49	0.30	23.6 ± 2.42	25.2 ± 3.0	0.08

Bivariable conditional logistic regression analyses were performed to assess the associations between active TB and thirteen potential predictor variables. These variables included drug use over the last year (OR, 4.88; 95% CI, 1.02–23.15), previous incarceration (OR, 6.82; 95% CI, 0.82–56.46), black race (OR, 3.71; 95% CI, 0.76–18.20), and serum 25(OH)D_3_ level <30 ng/mL (OR, 4.37; 95% CI, 1.56–12.25). In multivariable analysis, only subnormal serum 25(OH)D_3_ level (OR, 3.77; 95% CI, 1.04–13.64) was determined to be significantly associated with active TB after adjustment for drug use (OR, 2.87; 95% CI, 0.43–19.01), previous incarceration (OR, 3.12; 95% CI, 0.34–28.24) and black race (OR, 1.57; 95% CI, 0.26–9.71).

### Risk factors associated with TST conversion

In the first nested case-control study, we selected individuals who screened negative for TB at baseline by symptoms and sputum culture, and compared those who converted their TST from negative to positive when retested at one year (converters) with those who remained negative upon repeat testing (non-converters). The mean age of the converters was 32 years (±6 years) and 31 years (±7 years) among the non-converters (Table 2). Vitamin D levels did not differ significantly between the TST converters (37.7 ± 11.93 ng/mL) and non-converters (34.5 ± 14.89 ng/mL, p = 0.19). Albumin levels differed significantly between cases (4.63 ± 0.43 g/dL) and controls (4.28 ± 1.00 g/dL, p < 0.01) but were in the normal range (>3.5–5.2 g/dL) (Table 2). All cases and controls had negative HIV serology (Table 1).

In bivariable analysis, black race (OR, 3.12; 95% CI, 0.98–9.95), less than 4 years of schooling (OR, 2.44; 95% CI, 1.01–5.94), cigarette smoking (OR, 0.44; 95% CI, 0.17–1.15), serum 25(OH)D_3_ level <30 ng/mL (OR, 0.79; 95% CI, 0.31–2.04) and serum albumin level (OR, 2.05; 95% CI, 0.97–4.32) were associated with risk of TST conversion. The multivariable analysis showed black race (OR, 11.52; 95% CI, 2.01–63.36), less than 4 years of schooling (OR, 2.70; 95% CI, 0.90–8.16), cigarette smoking (OR, 0.22; 95% CI, 0.06–0.79) and serum albumin level (OR, 0.09; 95% CI, 0.01–1.13) were associated with an increased risk of TST conversion (Table 3). Vitamin D levels, however, were not associated with risk of TST conversion (OR, 2.49; 95% CI, 0.64–9.66; Table 3).

**Table 3:** Results of the bivariable and multivariable analyses of potential risk factors for active TB, TST conversion and progression to active TB in prisoners in Mato Grosso do Sul. Variables statistically significant in the multivariable analysis: *p < 0.05; **p < 0.01. TB, tuberculosis; TST, tuberculin skin test; OR, odds ratio; CI, confidence interval; NA, not applicable.

Variables	Case-control study		Nested case-control study		Nested case-control study	
	*Active TB*		*TST conversion*		*Progression to active TB*	
	Bivariable analysis OR (95% CI)	Multivariable analysis OR (95% CI)	Bivariable analysis OR (95% CI)	Multivariable analysis OR (95% CI)	Bivariable analysis OR (95% CI)	Multivariable analysis OR (95% CI)
Age > 30 years	0.72 (0.18–2.88)		0.46 (0.08–2.75)		1.00 (0.27–3.72)	
Marital status (single)	1.53 (0.56–4.16)		1.46 (0.57–3.77)		1.24 (0.40–3.81)	
Black race	3.71 (0.76–18.20)	1.57 (0.26–9.71)	3.12 (0.98–9.95)	11.52 (2.01–63.36)**	3.37 (1.01–11.18)	3.43 (0.85–13.75)
Resided in MS	0.91 (0.32–2.57)		1.17 (0.45–3.07)		1.00 (0.33–3.04)	
Less than 4 years of schooling	2.10 (0.52–8.43)		2.44 (1.01–5.94)	2.70 (0.90–8.16)	1.12 (0.35–3.61)	
Cigarette smoking	2.14 (0.68–6.74)		0.44 (0.17–1.15)	0.22 (0.06–0.79)*	7.77 (1.68–35.86)	7.42 (1.23–44.70)*
Drug use during the last year	4.88 (1.02–23.15)	2.87 (0.43–19.01)	0.91 (0.33–2.51)		4.28 (0.90–20.30)	2.00 (0.38–10.44)
Alcohol use	1.65 (0.58–4.69)		0.49 (0.17–1.38)		1.00 (0.33–3.04)	
Previous TB	2.38 (0.38–14.97)		1.00 (0.09–11.03)		2.00 (0.40–9.91)	
BCG Scar	0.37 (0.08–1.68)		0.57 (0.18–1.77)		0.59 (0.14–2.41)	
Previously incarcerated	6.82 (0.82–56.46)	3.12 (0.34–28.24)	0.75 (0.30–1.86)		0.75 (0.26–2.15)	
TST positive	1.39 (0.57–3.38)		NA		0.66 (0.26–1.64)	
Body Mass Index < 25	NA		1.22 (0.41–3.59)		2.53 (0.50–12.65)	
Serum 25(OH)D level < 30 ng/mL	4.37 (1.56–12.25)	3.77 (1.04–13.64)*	0.80 (0.31–2.05)	2.49 (0.64–9.66)	1.13 (0.34–3.75)	0.59 (0.13–2.62)
Serum albumin level < 3.5 g/dL	NA		0.17 (0.02–1.51)	0.09 (0.01–1.13)	1.82 (0.28–11.95)	

### Risk factors associated with progression to active TB

In the second nested case-control study, we compared baseline characteristics of individuals, who screened negative for active TB at baseline, according to whether they progressed to active TB over the subsequent year (“progressors”) or did not (“non-progressors”). Thirty-six percent of those who progressed and 50% of those who did not progress had positive TSTs at baseline. No significant difference in baseline serum 25(OH)D_3_ levels was identified between progressors (37.0 ± 14.83 ng/mL) and non-progressors (37.5 ± 11.04 ng/mL, p = 0.86; Table 2). All cases had negative HIV serology and only one control had positive HIV serology (Table 1).

In bivariable analysis, drug use during the last year (OR, 4.28; 95% CI, 0.90–20.30), cigarette smoking (OR, 7.77; 95% CI, 1.68–35.86) and black race (OR, 3.37; 95% CI, 1.01–11.18) were associated with risk of TB progression. However, in the multivariable analysis, only current smoker (OR, 7.42; 95% CI, 1.23–44.70) was associated with the development of active TB after adjustment for black race (OR, 3.43; 95% CI, 0.85–13.75), drug use during the last year (OR, 2.00; 95% CI, 0.38–10.44), and serum 25(OH)D_3_ level <30 ng/mL (OR, 0.59; 95% CI, 0.13–2.62; Table 3).

The average time to active TB diagnosis among progressors was 6.9 months. No statistical difference in serum 25(OH)D_3_ level at enrollment was observed among prisoners who progressed to active TB in the first 6 months compared to prisoners who had progressed after 6 months of follow-up (35.5 ng/mL ±12.01 and 38.4 ng/mL ±15.36, respectively; p = 0.46).

## Discussion

Despite the low power, this is the largest study ever published that evaluate the association of vitamin D levels with progression to TST conversion and progression of active disease in the prison population. We found that prisoners with active TB had significantly lower vitamin D levels at the time of diagnosis compared with prisons without active TB. However, baseline vitamin D levels were not predictive of TST conversion or progression to active disease. Notably, while 75% of prisoners with active TB had subnormal vitamin D levels at TB diagnosis, less than 33% of all other groups of participants (cases and controls) presented subnormal levels. These findings provide support for a pattern of disrupted vitamin D metabolism that results from the presence of active TB disease, and do not provide evidence for subnormal vitamin increasing susceptibility to infection or disease.

Our primary findings of poor vitamin D status associated with active TB among prisoners were consistent with the results of case-control studies conducted in the general population^8,17–26^. Furthermore, previous cohort studies conducted among household contacts of TB cases have suggested that low levels of vitamin D may be associated with risk of subsequent *M*. *tuberculosis* infection^27^ and development of active TB^28,29^; we did not find these associations in this study when vitamin D levels were examined months prior to diagnosis and found to be largely normal. The number of subjects experiencing the outcome of interest in previous studies was limited, however, ranging from 3 to 18 over follow-up periods 2 to 4 years. These studies have been found to vary with study design, geographical location^8^, as well as ethnicities^30^ and gender^24^ of subjects. Thus, studies with larger sample sizes in different populations are needed to help clarify whether the association between vitamin D and susceptibility to TB infection or disease is causal in nature.

Notably, we previously identified high rates of conversion (28%) and incident active disease (1.39%) over one year in this cohort study^10^. These rates provided more robust sample sizes of 30 and 24 cases, respectively, for the nested case-control studies to examine the impact of vitamin D status at baseline. Surprisingly, the majority of healthy prisoners show normal vitamin D. In the individuals without active disease at enrollment, both diet and sun exposures in this incarceration setting appear to be adequate to sustain precursor vitamin D levels in healthy prisoners. Vitamin D status under these conditions appeared not to be associated with either the acquisition of latent infection, or the development of active disease in this prison population. It is possible that there was insufficient heterogeneity in vitamin D in this population and setting to detect such effects. Here, factors other than vitamin D levels (e.g. smoking status) appear to contribute risk along the TB disease pathway.

Previous studies have recognized that inflammatory process may lower 25(OH)D_3_ levels as the precursor supply for activated vitamin D^31,32^. The normal immune response against *M*. *tuberculosis* produces inflammatory cytokines that activate CYP27B1 (1α-hydroxylase), converting available 25(OH)D_3_ to its activated form 1,25(OH)_2_D_3_^31,33^ and contributing to the measurement of low 25(OH)D_3_ levels^34^. Thus, the low levels of 25(OH)D_3_ (precursor) identified among prisoners with active TB in our case-control and among in several previous studies may have been associated with high conversion rates to 1,25(OH)2D_3_ caused by persistent disease. Notably, elevated plasma 1,25(OH)2D3 levels have been found in TB patients compared to healthy individuals^35,36^, suggesting hyperresponsive metabolism to form activated vitamin D may be associated with the disease in humans. However, vitamin D supplementation as part of randomized clinical trials during TB treatment has not been shown to reduce time to sputum culture conversion after 8 weeks^37–44^.

One of the major defense mechanisms involving vitamin D and *M*. *tuberculosis* is associated with Toll-like receptor (TLR)^45^. Mycobacterial antigens, especially lipoproteins, are recognized by TLR2 or associated with TLR1 or TLR6 which when activated participate in the expression of vitamin D receptor (VDR) and activation of 25(OH)D, assisting in the release of cathelicidin, a peptide capable of inhibiting the intracellular growth of *M*. *tuberculosis*, *in vivo*^46^ and *in vitro*^45^.

Vitamin D levels may be influenced by anti-TB drugs, especially rifampicin and isoniazid^47^, and if vitamin D measurement is performed after the initiation of treatment, these levels may be considerably lower among patients with TB^17,48–51^. One strength of our study was that all serum samples used to measure vitamin D levels were collected before treatment was initiated removing this potential factor impacting vitamin D.

Risk of TST conversion was not influenced by baseline vitamin D status in the prisoners, but other risk factors showed associations consistent with the previous reports. For example, previous studies reported an association between being a current smoker and TB^52,53^. Evidence suggests that smoking decreases TST reactivity^54,55^ but increases the risk of progression to active TB^56^ by directly impairing host immunity, especially the responses of T-cells and macrophages to *M*. *tuberculosis*^57,58^. An impaired immune response makes it more difficult to eliminate *M*. *Tuberculosis* and easier to develop the active disease^52,57^. While the variable cigarette smoking was found here to exert a protective effect on risk of TST conversion to latent infection, it dramatically increased risk of progression to active TB. TST conversion was also associated with black race, which increased the odds of infection by *M*. *tuberculosis*.

Evidence suggests that black race may increase risk of *M*. *tuberculosis* infection greater than other racial backgrounds^59^. Although the mechanism underlying this association has not been fully elucidated, it may be attributed to environmental factors, unfavorable socioeconomic conditions^60,61^, and genetic factors^62,63^. Black skin can impair vitamin D synthesis from UVB exposure, but notably, vitamin D status was found to be an independent risk factor for active TB when controlling for race in the case-control study. Although it is not yet clear why race is a risk factor for TST conversion, we believe that in addition to the polymorphisms vitamin D receptors, other genetic factors may be associated. In our study, in the final model, the level of Vitamin D was adjusted by race and even then, the serum level of vitamin D was not associated with TST conversion. Other polymorphisms associated with black race should be investigated.

Low levels of schooling also showed a tendency toward increased risk and may be directly related with incarceration mixing patterns of high risk individuals during incarceration^64^. The prisoners are combined in cell blocks of mixed socioeconomic conditions, thus increasing the probability of becoming infected with *M*. *tuberculosis*^65,66^. However, the fact that all prisoners were exposed to the same conditions during incarceration, reinforces the need for further studies to be conducted to explain the reasons behind the racial and education inequalities among prisoners with active TB.

We examined risk of incident TB among all prisoners rather than those who were TST positive at baseline. The majority of TB disease in this high transmission setting occurs due to recent infection rather that late reactivation. Indeed, the majority of incident TB cases over one year in this cohort occurred among individuals who were TST negative at baseline.

While studies remain challenged by power, this work represents the largest study ever conducted to evaluate TST conversion and progression of active disease. We used the TST conversion to assess TB infections and progression to active TB, however, some studies indicate that TST can cause false-positive results by BCG vaccination and exposure to non-tuberculosis mycobacteria (NTM). In Brazil, BCG vaccination usually performed in the first days of life that does not interfere with the TST result if carried out 10 years or more after vaccination^67,68^. In this study the average age was 32.1 (±6.87, p = 0.50) in TST conversion and 30.8 (±8.90, p = 0.63) in progression to active TB, it seems unlikely that BCG vaccination interfered with the TST results. As well as, exposure to non-tuberculosis mycobacteria (NTM) may interferes with TST results only in populations with a high prevalence of NTM sensitization and low TB infection, that does not happen in our study population, where the incidence of active TB is at least 30 times higher than that observed in the general population^10^. Interferon-ɤ release assays (IGRA) to determinate TST conversion may be more useful in populations where the BCG vaccine has not been administered or if the test was performed within 10 years of vaccination, thus, in our study, the TST and IGRAs likely would generate similar findings^67^.

Data suggest that seasonality^69^ and the incarceration time^70^ may favor a decrease in vitamin D levels. A strength of our design however, was that all prisoners were screened in the same months, and cases and controls were paired according to the prison cell block and incarceration time, having the same time of potential sun exposure and similar diet; these features help minimize confounding in our analysis, however is very difficult to perfectly match due to the prolonged and variable infectious period, variable social mixing, and frequent movement of inmates between cells. Overmatching among cases and controls, may have decreased the variability in vitamin D levels limiting the ability to identify important associations over the spectrum of vitamin D with the disease pathway. As a caveat, as many participants have sufficient vitamin D this may play a minor role in relation to other risk factors.

## Conclusion

We identified that prisoners with active TB had poor vitamin D status; however, vitamin D levels were not identified to be a risk factor for TST conversion or the development of active TB over a follow-up period of one year in the evaluated prison population. Other risk factors such as black race and low education were found to be risk factors for TST conversion, while smoking was the only variable associated with disease progression. Our findings suggest active TB may alter vitamin D metabolism, leading to compromised precursor supply, but that vitamin D status is not necessarily a risk factor for susceptibility to infection or disease.

## Methods

### Population

In 2013, the state of Mato Grosso do Sul (MS), located in the west-central Brazil, had the country’s seventh largest prison population, including 14,904 prisoners, and the higher rate of national incarceration (568.9 prisoners per 100,000 inhabitants)^71^. A one-year prospective cohort study was conducted in 2013 in 12 prisons (8 male prisons, with 6,552 prisoners and 4 female prisons, with 669 prisoners) to identify the incidence of latent and active TB^10,16^. The case-study and two nested case-studies here are part of this larger cohort study.

Prisoners who were over 18 years old and consented to participate in the study were recruited from a randomly stratified sample at the 12 prisons. A structured questionnaire was administered and a (TST) (PPD RT23, Staten Serum Institute, Copenhagen) was performed. Two sputum samples were obtained from all participants reporting cough, and smear microscopy and culture, using the Ogawa-Kudoh technique, was performed. Although X-ray screening shows good sensitivity, and is an important method for screening suspected cases of TB, the specificity of X-ray screening is low. Additionally, chest radiography is not available in the study prisons. For these reasons, we used sputum culture to define TB cases. Individuals with at least one smear or culture result positive for *M*. *tuberculosis* were considered TB cases. A venous blood sample was collected, and plasma was frozen for assessment of vitamin D levels.

TST positivity was defined as an induration of ≥10 mm measured by a trained TST reader, 48 hours after injected. After one year, in 2014, a second TST was performed, and two sputum samples were collected for smear microscopy and culture from all participants reporting cough at the second time point. TST conversion was defined as TST ≥10 mm and an induration increase of at least 6 mm in an individual with a baseline TST <10 mm^10^.

Incident TB cases were defined as those occurring after the first screening time point, including those diagnosed between the two screening dates (day 0 and day 365) and those diagnosed on the second screening date. Those diagnosed between the screening dates were diagnosed through routine medical services provided by the prison, in which symptomatic individuals are clinically evaluated and sputum is sent for smear and/or culture as determined the prison clinician. These TB cases occurring between the screening dates were identified through prison medical records and the National Notifiable Disease database (Sistema de Informação de Agravos de Notificação National, SINAN).

A plasma sample for vitamin D levels was collected at the time of enrollment, which was prior to TB diagnosis, and therefore, prior to initiation of treatment. Due to the low rates of TB in the female prisoners, the vitamin D evaluation was conducted in only male prisoners^10^.

All eligible participants provided written informed consent prior to study participation. All methods described in this study were approved by Research Ethics Committee of the Federal University of Grande Dourados (Number 191,877 and 793,740) and were performed in accordance with the relevant guidelines and regulations. The study was authorized by the State Agency of Administration of the Penitentiary System (AGEPEN) who gave full support during the study period.

### Risk factors associated with active TB

**Figure1:**
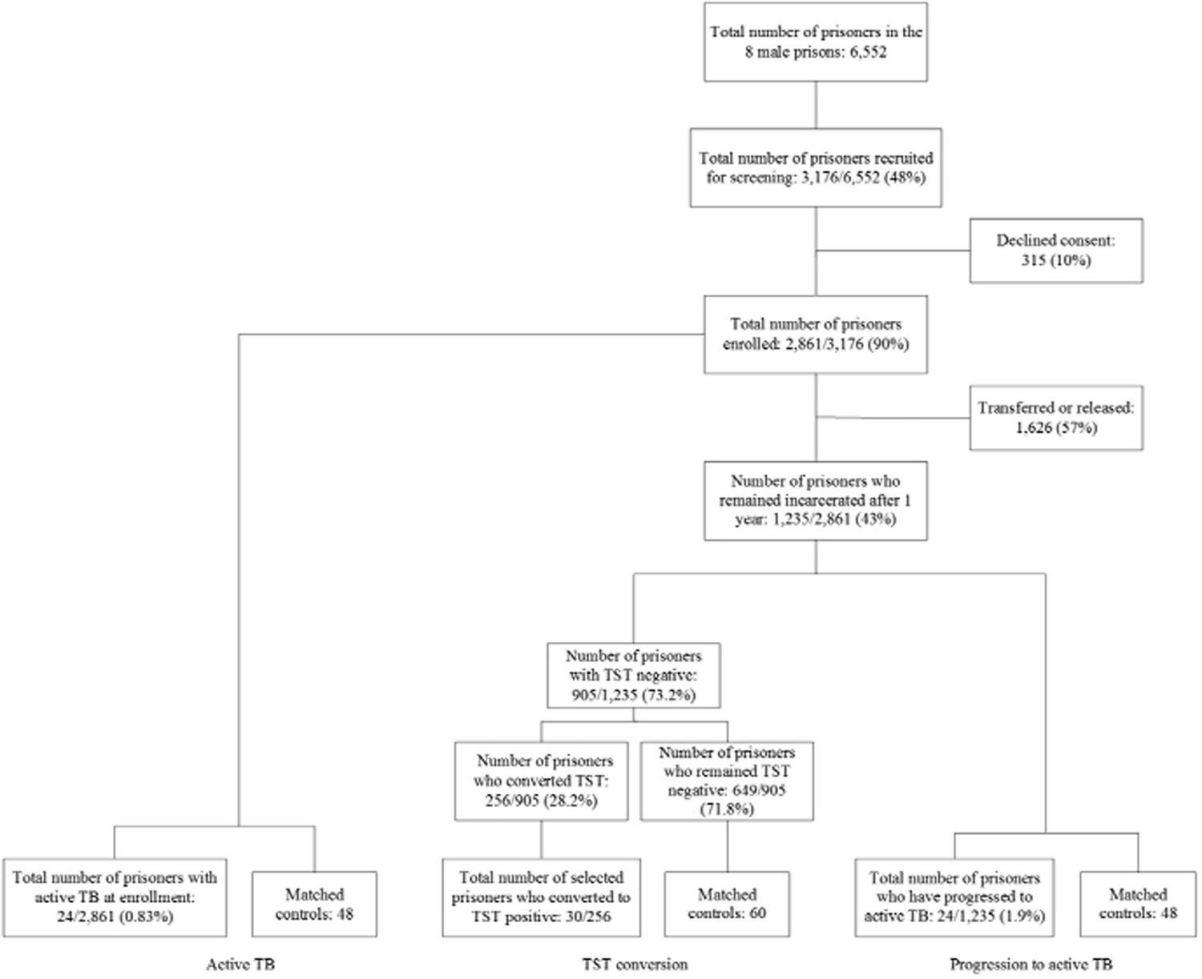
Flow chart of the study process in 8 male prisons in Mato Grosso do Sul.

During enrollment in the cohort study, a total of 29 male prisoners from 2,861 were culture positive for *M*. *tuberculosis* and, therefore, had active TB^16^ (Fig. 1). Based on this finding, a case-control study was performed in which 25(OH)D levels were measured in serum samples obtained from 24 prisoners with active TB and compared with those from samples obtained from 48 controls, 25 of whom were TST-negative and 23 were TST positive, who were matched on the basis of age (±5 years), prison cell block and incarceration time (±6 months) (Fig. 1). Other risk factors of interest were age, marital status, education, resides in MS, smoking, drug use over the last year, alcohol, contact with a TB-positive individual and previous incarceration. The participant’s race/skin color (i.e., white, black, indigenous, asian or mixed) was self-reported. The presence of a Bacillus Calmette-Guérin (BCG) vaccine scar was determined by inspecting the participant’s arm. The body mass index (BMI) wasn’t included in evaluation of active TB because we do not know if cause or effect.

### Risk factors associated with TST conversion

A nested case-control study was performed to identify risk factors associated with TST conversion. Thirty prisoners randomly who converted their TST from negative to positive (converters) over the course of 12 months were compared with sixty prisoners who had negative TST tests at baseline and repeat testing at 12 months (non-converters) (Fig. 1). Controls were matched based on age (±5 years), prison cell block and incarceration time (±6 months). Other risk factors assessed for TST conversion were age, marital status, education, resides in MS, smoking, drug use over the last year, alcohol, serum albumin, contact with a TB-positive individual, BMI and previous incarceration. The presence of a BCG vaccine scar was determined by inspecting the participant’s arm.

### Risk factors associated with progression to active TB

A nested case-control study was conducted to investigate the association between vitamin D levels and risk of subsequent TB disease. Of the 1,235 prisoners who remained incarcerated after 1 year in the male prison system (Fig. 1), we selected 24 individuals without TB at baseline who were diagnosed with active TB over the course of 12 months (progressors) and compared them with 48 individuals who were TB negative throughout 12 months (non-progressors), as documented by active TB screening at baseline and 12 months. Controls were matched to cases on the basis of age (±5 years), prison cell block and incarceration time (±6 months) (Fig. 1). The risk factors assessed for progression to active TB were age, marital status, education, resides in MS, smoking, drug use over the last year, alcohol, serum albumin, contact with a TB-positive individual, BMI and previous incarceration. The presence of a BCG vaccine scar was determined by inspecting the participant’s arm.

### Biochemical analyses

Vitamin D levels were measured in serum samples, which were stored away from light and at −20 °C until the time of analysis. Levels of 25(OH)D (ng/ml) were identified using the automated electrochemiluminescence immunoassay method and Cobas® e411 analyzer (Roche Diagnostics, Mannheim, Germany). Serum 25(OH)D levels were considered normal when values were ≥30 ng/mL^72–74^. Subnormal vitamin D was used to indicate combined groups for vitamin D insufficiency (29–20 ng/ml) and deficiency (<20 ng/ml). Serum albumin levels were measured in samples obtained from prisoners included the nested case-control studies using the Cobas Integra® 400 plus analyzer (Roche Diagnostics, Mannheim, Germany). All procedures were performed in the Laboratory of the University Hospital of the Federal University of Grande Dourados.

### Data analysis

All questionnaires were double entered into the Research Electronic Data Capture database (REDCap). SAS version 9.2 (SAS Institute, Cary, NC, USA) was used to perform the analyses. We used the t-test for continuous variables, Chi-square test for categorical variables and bivariable conditional logistic regression analyses to examine the associations between dependent and independent variables. Vitamin D insufficient and all variables with p ≤ 0.10 in the bivariable analyses were included in multivariable conditional logistic regression analysis. P values ≤0.05 were considered statistically significant.

### Availability of materials and data

The data will not be shared in order to protect the participants’ anonymity.
